# *GJB*2 and *GJB*6 Genetic Variant Curation in an Argentinean Non-Syndromic Hearing-Impaired Cohort

**DOI:** 10.3390/genes11101233

**Published:** 2020-10-21

**Authors:** Paula Buonfiglio, Carlos D. Bruque, Leonela Luce, Florencia Giliberto, Vanesa Lotersztein, Sebastián Menazzi, Bibiana Paoli, Ana Belén Elgoyhen, Viviana Dalamón

**Affiliations:** 1 Laboratorio de Fisiología y Genética de la Audición, Instituto de Investigaciones en Ingeniería Genética y Biología Molecular “Dr. Héctor N. Torres”, Consejo Nacional de Investigaciones Científicas y Técnicas—INGEBI/CONICET, C1428ADN Ciudad Autónoma de Buenos Aires, Argentina; paulabuonfiglio@gmail.com (P.B.); abelgoyhen@gmail.com (A.B.E.); 2 Centro Nacional de Genética Médica, ANLIS-Malbrán, C1425 Ciudad Autónoma de Buenos Aires, Argentina; bruquecarlos@gmail.com; 3 Instituto de Biología y Medicina Experimental, Consejo Nacional de Investigaciones Científicas y Técnicas—IBYME/CONICET, C1428ADN Ciudad Autónoma de Buenos Aires, Argentina; 4 Laboratorio de Distrofinopatías, Cátedra de Genética, Facultad de Farmacia y Bioquímica, Universidad de Buenos Aires, C1113AAD Ciudad Autónoma de Buenos Aires, Argentina; leonelaluce@gmail.com (L.L.); gilibertoflor@gmail.com (F.G.); 5 Instituto de Inmunología, Genética y Metabolismo—INIGEM/CONICET, Universidad de Buenos Aires, C1113AAD Ciudad Autónoma de Buenos Aires, Argentina; 6 Servicio de Genética, Hospital Militar Central “Dr. Cosme Argerich”, C1426 Ciudad Autónoma de Buenos Aires, Argentina; vlotersztein@yahoo.com.ar; 7 Servicio de Genética, Hospital de Clínicas “José de San Martín”, C1120AAR Ciudad Autónoma de Buenos Aires, Argentina; smenazzi@gmail.com; 8 Servicio de Otorrinolaringología Infantil, Hospital de Clínicas “José de San Martín”, C1120AAR Ciudad Autónoma de Buenos Aires, Argentina; bibianapaoli@uolsinectis.com.ar; 9 Departamento de Farmacología, Facultad de Medicina, Universidad de Buenos Aires, C1121ABG Ciudad Autónoma de Buenos Aires, Argentina

**Keywords:** *GJB*2, *GJB*6, genetic variants, curation, hearing loss, argentina

## Abstract

Genetic variants in *GJB*2 and *GJB*6 genes are the most frequent causes of hereditary hearing loss among several deaf populations worldwide. Molecular diagnosis enables proper genetic counseling and medical prognosis to patients. In this study, we present an update of testing results in a cohort of Argentinean non-syndromic hearing-impaired individuals. A total of 48 different sequence variants were detected in genomic DNA from patients referred to our laboratory. They were manually curated and classified based on the American College of Medical Genetics and Genomics/Association for Molecular Pathology ACMG/AMP standards and hearing-loss-gene-specific criteria of the ClinGen Hearing Loss Expert Panel. More than 50% of sequence variants were reclassified from their previous categorization in ClinVar. These results provide an accurately interpreted set of variants to be taken into account by clinicians and the scientific community, and hence, aid the precise genetic counseling to patients.

## 1. Introduction

Congenital hearing loss (HL) is the most common sensory disorder that affects approximately 1–2 of 1000 infants, with 50% of cases resulting from genetic factors [1]. In 70–80% of neonates who fail newborn hearing screening, no other distinguishing physical findings are present and the HL is classified as non-syndromic. The majority of non-syndromic cases are of autosomal recessive inheritance (80%), 12–15% autosomal dominant, 1–5% X-linked and 1–5% mitochondrial [2]. In general, autosomal recessive loci are related to a prelingual HL, while autosomal dominant loci to a postlingual HL phenotype [3]. A large number of genes are involved in hereditary HL. To date, a total of 121 non-syndromic causative genes have been described: 76 of recessive inheritance, 49 of dominant inheritance and five X-linked (some genes can cause recessive and dominant hearing impairment) [4]. This landscape illustrates the auditory system complexity, comprising a large number of proteins, which together participate in hearing physiology and development [5].

Despite the wide genetic heterogeneity of hearing impairment, the most commonly mutated genes in severe to profound autosomal recessive non-syndromic hearing loss (ARNSHL) are *GJB*2 and *GJB*6 (encoding connexin-26 and 30, respectively), accounting for nearly 50% of the cases in most populations around the Mediterranean Sea [6,7,8,9,10,11]. *GJB*2 and *GJB*6 (DFNB1) genes are part of a gene family that encode gap-junction proteins. It is well demonstrated that they are expressed in cochlear supporting cells, with a role in endolymph potassium recycling, inositol triphosphate (IP3) transfer and diffusion of different metabolites [12,13]. Connexins (Cx) are formed of four transmembrane domains, two extracellular loops and three cytoplasmic domains: the amino-terminus, a cytoplasmic loop and the carboxy-terminus domain [13].

Both *GJB*2 and *GJB*6 are located in chromosome 13q12. The *GJB*2 gene comprises two exons and the coding region is completely contained in the second exon, leading to a 2290-nucleotide mRNA (GeneBank: NM_004004.6). On the other hand, the *GJB*6 gene consists of five exons and the last one contains the entire coding sequence which is transcribed to a 2110 bp mRNA (GeneBank: NM_006783.4). In general, mutations which produce a loss of gap-junction channel function are related to non-syndromic ARSHL [14,15,16].

The most frequent mutation in *GJB*2 is c.35delG in the Caucasian population [7,9,17,18,19]. In addition, there are more than 300 pathogenic variants identified in *GJB*2 (Deafness Variation Database) [20]. In the case of *GJB*6, two large deletions of 309 and 232-kb, del(*GJB*6-D13S1830) and del(*GJB*6-D13S1854), respectively, in the 5′ region of the gene, along with other rarer and less studied deletions, have been described [10,21,22,23,24,25]. Previous studies have demonstrated that different cohorts of Argentinean patients carry similar frequent genetic variants in *GJB*2 and *GJB*6 [26,27,28,29,30,31].

Identifying the genetic etiology of hearing impairment can provide proper counseling, clinical management and accurate estimation of deafness odds recurrence within a family [29,30]. Moreover, molecular diagnosis contributes with valuable prognosis information: DFNB1-hearing loss is not related to other phenotypic symptoms nor to significant hearing loss progression over time, and in general, it is related to a congenital profound bilateral hearing loss [32]. Affected probands carrying two truncating/nonsense variants in *GJB*2 present a more severe degree of hearing loss than those who carry two missense variants [18,28,33,34]. Furthermore, patients with *GJB*2 genetic variants present excellent outcomes in speech perception/production skills after cochlear implantation [35,36,37]. Therefore, correct interpretation of the phenotypic consequences of genetic variants is crucial in genetic diagnosis, since discrepancies in sequence variant interpretation and classification has been reported to lead to serious impact in patient health maintenance [38,39,40]. Thus, the American College of Medical Genetics and Genomics (ACMG) and the Association for Molecular Pathology (AMP) has developed guidelines for clinical interpretation of genetic variants [41]. In addition, the ClinGen Hearing Loss Clinical Domain Working Group (HLWG) has adapted the ACMG/AMP guidelines for the classification of genetic variants in the hearing loss framework [28,42].

In the present study we aimed to identify causative mutations in *GJB*2 and *GJB*6 genes in Argentinean non-syndromic hearing-impaired patients and report an update of allele and genotype frequencies. Furthermore, we performed a thorough manual curation of sequence variants according to ACMG/AMP standards and applied rigorously the latest hearing loss gene-specific criteria of the ClinGen Hearing Loss Expert Panel (HL-EP) [41,42]. These findings clearly highlight the importance of genetic studies with the appropriate comprehensive analysis by experts in the field, with the goal of providing an accurate molecular diagnosis, and consequently, precise genetic counseling to the patients.

## 2. Materials and Methods

### 2.1. Part A: Identification of Variants in an Argentinean Cohort

#### 2.1.1. Patients

**Figure 1:**
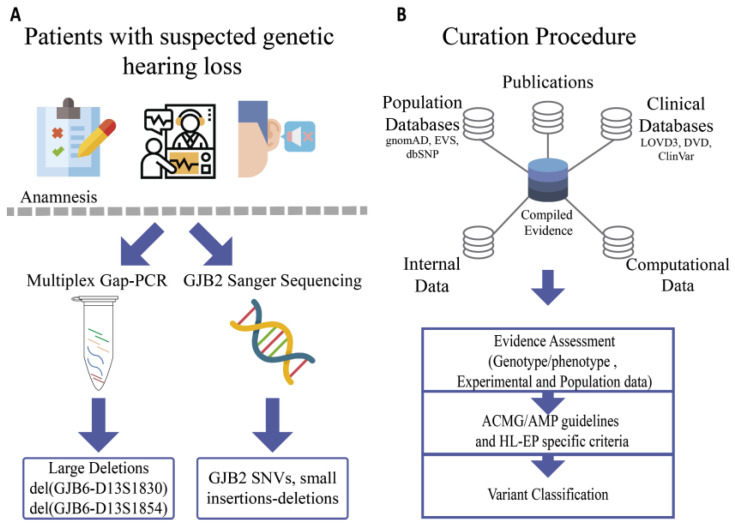
General workflow of this study. (**A**): Molecular screening of patients. Some icons were obtained from flaticon webpage [67]. (**B**): Variant curation process.

This study includes a total of 600 Argentinean non-related patients (290 females and 310 males) with non-syndromic sensorineural hearing loss. Clinical evaluation was performed by a clinical geneticist and included: personal history, physical examination, audiometric information, age of hearing impairment onset, hearing thresholds, pedigree and genetic assessment. For each patient, a complete medical history was obtained to exclude the possibility of environmental causes of hearing impairment (e.g., ototoxic drugs, infectious diseases, acoustic trauma). All subjects gave their informed consent for inclusion before they participated in the study. The study was conducted in accordance with the Declaration of Helsinki, and the protocol was approved by the Ethics Committee of Administración Nacional de Laboratorios e Institutos de Salud (ANLIS) (1912–2018). The workflow is summarized in Figure 1A.

A total of 477 sporadic and 123 familial cases (80% and 20%, respectively) were sequentially referred to the Laboratory of Physiology and Genetic of Hearing, INGEBI, in Buenos Aires, Argentina from 2004 to March 2020. Familial cases were of a dominant and recessive form of inheritance (59 and 64/123). All patients were analyzed by an ear-nose-throat (ENT) specialist using standard methods. The severity of deafness was classified considering the following thresholds in decibels: mild (20 to 39 dB), moderate (40 to 69 dB), severe (70 to 89 dB) and profound (90 dB). The patient’s deafness severity was defined by the ear with the minor degree of hearing loss. Complete audiological history data were compiled from affected subjects in case of need. Overall, 479 of patients (47%) exhibited prelingual HL, while 121 (20%) a postlingual phenotype. The severity of HL was: 102 moderate, 106 severe and 392 profound. A total of 69 patients were cochlear implanted. This prospective study (2004–2020) includes data previously reported in Dalamón et al. 2013 (*n* = 476 patients), but that was not curated following HL-EP standards. 

#### 2.1.2. Samples

Genomic DNA was isolated from whole blood samples extracted with 5% ethylene-diamine tetraacetic acid (EDTA) (Sigma-Aldrich, St. Louis, MO, USA) using the cetyltrimethyl-ammonium bromide (CTAB) (Sigma-Aldrich, St. Louis, MO, USA) method [43]. DNA concentration and quality were measured by absorbance at 260 nm and by the A260 nm/A280 nm and A260 nm/A230 nm ratios, respectively (NanoDrop^TM^) (Thermo Fisher Scientific, Wilmington, NC, USA). All samples were stored at −20 °C.

#### 2.1.3. *GJB*2/*GJB*6 Molecular Studies

Genetic variants in *GJB*2 were studied by direct sequencing of the coding exon 2, non-coding exon 1 and intronic boundaries. The splice site variants c.-23+1G>A and c.-22-2A>C were included in the screening. Primers, protocols and cycling programs used were as previously reported [28]. Bidirectional DNA sequencing was performed on an automatic sequencer (3730xl DNA Analyzer, Applied Biosystems, Foster City, CA, USA). Sequences obtained were analyzed by CodonCodeAligner program [44] and the BLAST NCBI interface (Basic local alignment search tool) [45] using the consensus sequence of *GJB*2 gene (GeneBank NG_008358.1). To examine the large deletions in *GJB*6:del(*GJB*6-D13S1830) and del(*GJB*6-D13S1854), a GAP-PCR and subsequent analysis were performed according to reported protocols [10,21].

#### 2.1.4. Data Analysis

In order to establish a genotype/phenotype correlation, presumably pathogenic identified *GJB*2 allele variants were classified as truncating (T) and non-truncating (NT) mutations [46]. Truncating mutations are loss-of-function (LoF) and include nonsense variants, insertions, deletions and duplications that introduce a shift in reading frame leading to a premature termination of protein translation, as well as the donor splice-site variant c.-23+1G>A leading to non-functional mRNA. Both del(*GJB*6-D13S1830) and del(*GJB*6-D13S1854) were also classified as truncating, because they lead to a nearly complete absence of Cx26 protein expression [10,47,48]. The group of non-truncating variants consists of missense variants (leading to amino acid substitutions) and the in-frame deletion (delGlu120). The acceptor splice-site variant c.-22-2A>C was defined as non-truncating since a residual expression of the wild type transcript due to the activation of an alternative acceptor splice site has been reported [49]. A chi square statistical analysis was performed in order to analyze the differences between groups.

### 2.2. Part B: Curation of Variants

#### *GJB*2-*GJB*6 Variant Curation

Manual curation of variants required information gathered from: population data, genotypes, segregation, phenotypic features and functional and experimental data of the reported variants. Nomenclature of sequence variants identified were achieved according to HGVS standards [50] and manually revised with the Mutalyzer name-checker tool [51]. Computational and predictive evidence was performed in silico through diverse strategies according to the type of variant analyzed: missense variants with REVEL [52] and Combined Annotation Dependent Depletion (CADD) [53] tools, splice site and silent variants with Human Splicing Finder [54] and MaxEntScan softwares [55] and loss of function variants (nonsense, frameshift and canonical splice site) following the ACMG/AMP recommendations [56]. REVEL, CADD and MaxEntScan scores were determined with the Variant Effect Predictor tool [57].

Reports in PubMed, as well as internal data from our laboratory, and seven different databases were used: 1. gnomAD [58], 2. dbSNP [59], 3. NHLBI-ESP’s EVS [60], 4. ClinVar [61], 5. LOVD [62], 6. Deafness Variation Database (DVD) [20] and 7. Database of Genomics Variants [63,64]. Variant filtering allele frequency was calculated by using inverse allele frequency [65]. More than 250 publications from PubMed were revised up to June 2020 to validate genetic variant interpretation. Clinical histories of patients were used to provide further information regarding segregation analysis and phenotypic features.

Collected information was manually assessed in order to organize and score the strength of evidence. Genetic variants were interpreted according to ACMG/AMP guidelines [41] and hearing loss gene specific criteria of the ClinGen HL-EP [42]. The final criteria score was manually assigned through the Varsome tool [66] in order to obtain the variant classification. Members of the Laboratory of Physiology and Genetics of Hearing discussed and reviewed final variant classification. A summary of information to be used by clinicians concerning variant specific criteria is detailed in Supplementary Table S1.

## 3. Results

### 3.1. General Genetic Findings

*GJB*2 and *GJB*6 single nucleotide variants (SNVs) and deletions del(*GJB*6-D13S1830) and del(*GJB*6-D13S1854) were studied in 600 NSHL Argentinean patients by Sanger Sequencing and GAP-PCR, respectively. Overall, 48 different sequence variants were identified in the 1200 alleles tested from the entire cohort of patients. The most frequent mutated alleles detected were: c.35delG (9.1%), p.Val27Ile (8.3%), p.Met34Thr (1.5%), c.167delT (1.16%) and the del(*GJB*6-D13S1830) (0.99%), followed by p.Val37Ile, p.(Glu47*), p.Arg143Trp, p.(Lys168Arg) and del(*GJB*6-D13S1854) with frequencies from 0.83% to 0.4%. Other variants were found less than five times in the cohort; their specific allele frequency is detailed hereafter.

**Figure 2:**
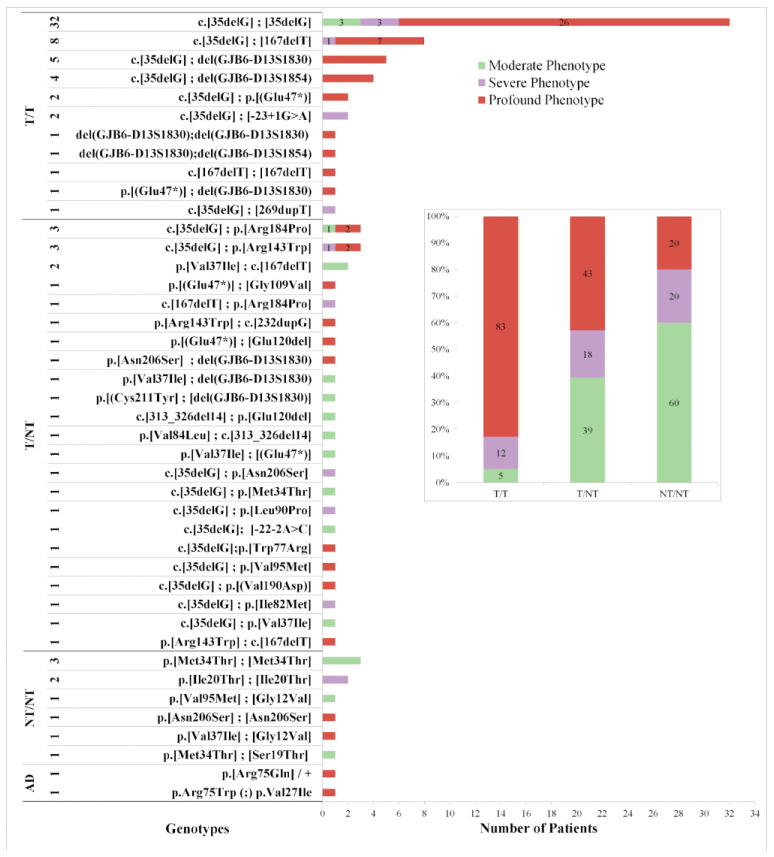
Distribution of Genotypes and Phenotypes of Patients. Moderate phenotype is shown in green, severe in violet and profound in red. The total number of each genotype is listed on the left together with its categorization: biallelic truncating (T/T), compound heterozygous truncating/non-truncating (T/NT) and biallelic non-truncating (NT/NT). Nomenclature was performed following HGVS recommendations; however, some variants keep the old annotation due to their common use in literature. In the inner box: Relative frequencies of the degree of HL in the three groups of genotypes. Biallelic T/T genotypes were mostly related to a worse degree of hearing impairment, since 83% of those patients exhibited profound HL. There were significant differences among the three groups, with X^2^ testing (*p* < 0.0001).

A total of 229 patients, representing 38% of the studied cohort, exhibited genetic variants in *GJB*2/*GJB*6, either in heterozygous (*n* = 117) or homozygous states, two different variants in the same gene, or *GJB*6 deletions in combination with *GJB*2 variants. Familial segregation was performed in 36/97 cases, confirming the in trans occurrence. All other genotypes involving two known causative variants were presumed of biallelic inheritance. A total of 42 diverse biallelic genotypes were identified (Figure 2). The most prevalent genotype detected was the homozygous c. (35delG) variant (33.3% of the biallelic mutations), followed by the compound heterozygous c.(35delG);(167delT) (8.33%). Genotype (*GJB*2:c.35delG);(*GJB*6:del(*GJB*6-D13S1830) and (*GJB*2:c.35delG);(*GJB*6:del(*GJB*6-D13S1854) were detected in 5.2% and 4.16% of hearing-impaired individuals, respectively. Compound heterozygous involving one of the large deletions in *GJB*6 accounted for the 15.6% of the total detected genotypes (15/96).

Biallelic causative mutations were found in 36% of ARNSHL familial cases (23/64) and 15.5% of sporadic ones (74/477). Overall, 38.5% of the cases were compound heterozygous for the c.(35delG) variant in trans with different mutations, and other 30.2% carried two different non-35delG variants.

Additionally, two patients carried the mutations p.Arg75Trp and p.Arg75Gln with a dominant mode of inheritance. A summary of genotypes, phenotypes and segregation is detailed in Supplementary Table S2.

### 3.2. Genotype-Phenotype Characterization

In order to correlate the identified genotypes with audiological features, we categorized the genetic variants as truncating (T) or non-truncating (NT). The variants of the 97 positively genotyped patients with biallelic recessive *GJB*2, dominant *GJB*2 and/or compound *GJB*2/*GJB*6 variants were correlated with their HL severity (moderate, severe, profound).

A total of 42 different genotypes were categorized: 11 homozygous truncating (T/T), 23 heterozygous truncating/non-truncating (T/NT), six homozygous non-truncating (NT/NT) and two autosomal dominant NT (AD). Distribution of genotypes/phenotypes and relative frequencies of the degree of HL in the three groups are shown in Figure 2.

Biallelic T/T genotypes were mostly related to a worse degree of hearing impairment, since 83% of those patients exhibited profound HL, 12% severe and 5% moderate HL. In contrast, biallelic NT/NT genotypes showed a milder degree of hearing impairment since 60% of these cases had moderate HL and 20% severe/profound HL. Compound heterozygous T/NT genotypes ranged from moderate to profound with no clear trends: 43% with profound HL, 18% severe HL and 39% moderate HL (Figure 2 inner box). There were significant differences among the three groups, with X^2^ testing (*p* < 0.0001).

### 3.3. Variant Curation

In order to further analyze and validate the identified variants, we performed a manual revision of available evidence following the Guidelines and recommendations of the ACMG/AMP and The ClinGen Hearing loss Expert Panel.

A total of 48 sequence variants were identified in *GJB*2 and *GJB*6 in our study cohort and 44 were manually curated, since four variants had been already curated as pathogenic by the HL-EP group: c.35delG, p.Met34Thr, p.Val37Ile and c.167delT. Variant c.-22-2A>C was reported as Variant of Unknown Significance (VUS) by the HL-EP group but was reclassified with new available information in this work explained further below.

From the total of variants analyzed, 23 were classified as pathogenic (P), three likely pathogenic (LP), nine uncertain significance (VUS), four likely benign (LB) and five benign (B). The final classification of the 44 variants and their general information is shown in Table 1.

**Figure 3:**
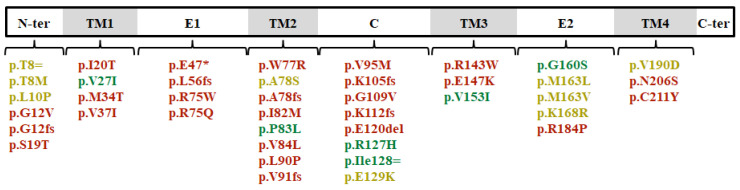
Distribution of coding genetic variants in connexin 26. Different colors refer to their classification after the curation process. Pathogenic and likely pathogenic variants are in red; benign and likely benign in green; uncertain significance in yellow.

Genetic variants’ distribution spanned the entire length of Cx26 and involved almost all protein domains (Figure 3).

**Figure 4:**
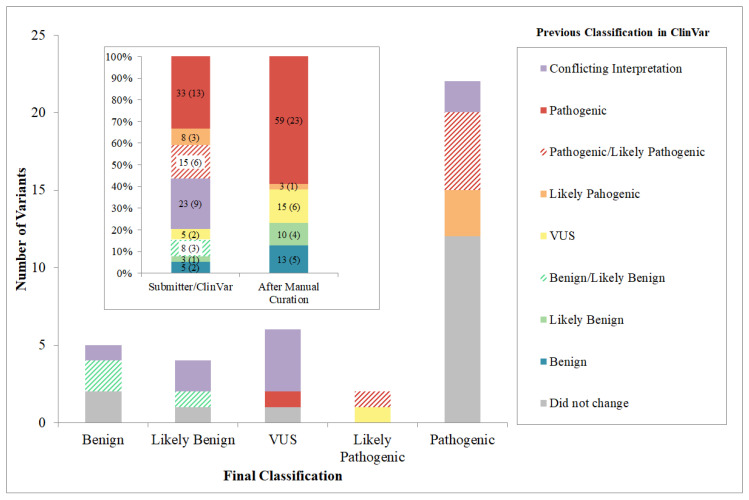
Final *GJB*2 and *GJB*6 variant classification. The height of each bar represents the number of variants for each classification. The colored segments of each bar represent previous classification in ClinVar. As a result of the curation process 59% of sequence variants changed their previous category submitted to ClinVar. The inner box shows the comparison of the 44 variant classifications between ClinVar submitters (from the original ACMG/AMP criteria) versus applying HL-EP specifications, demonstrating a reduction of “conflicting interpretation” and the increase of “pathogenic” categories. The number of variants for each interpretation is in brackets.

Interestingly, based on the specific criteria applied during our curation procedure, 59% of sequence variants evaluated in this study changed their previous category submitted to ClinVar: 36% with considerable re-interpretation and 23% with resolution of similar categories (for instance, P/LP submission for p.Gly12Val variant was confirmed as pathogenic or B/LB submission for p.Val153Ile variant was classified as benign) (Figure 4).

After the curation procedure, the pathogenic final classification represented a total of 23 variants, of which 11 changed their previous status in ClinVar, and 12 remained in the same category (Figure 4). In addition, nine variants previously considered as conflicting interpretation of pathogenicity were reclassified as: pathogenic (two cases), benign or likely benign (three cases) and uncertain significance (four cases). The c.269T>C, p.Leu90Pro and c.551G>C, p.Arg184Pro variants were reinterpreted as pathogenic, since both mutations had strong evidence concerning allelic data (PM3_VeryStrong) and functional studies demonstrating a deleterious effect (PS3). The c.380G>A, p.(Arg127His); c.478G>A, p.(Gly160Ser) and the 3′UTR variant c.*1C>T were reclassified to likely benign and benign mostly based on their high population frequencies (BA1 and BS1 rules). The last four variants with conflicting interpretations in ClinVar: c.23C>T, p.Thr8Met; c.24G>A, p.(Thr8=); c.385G>A, p.(Glu129Lys); c.503A>G, p.(Lys168Arg), were reclassified as being of uncertain significance since available information was not sufficient to determine their pathogenicity. The c.487A>C, p.Met163Leu variant listed in ClinVar as pathogenic for dominant HL, was reinterpreted in this work as being of uncertain significance, due to the lack of strong evidence: absent in population database (PM2), low REVEL score, functional study with only supporting evidence (PS3_P) and only reported three times (PS4_P).

Remarkably, we propose that the splice site variant c.-22-2A>C interpreted as being of uncertain significance by the HL-EP group should be reclassified as likely pathogenic according to new uncovered data. Thus, due to the usage of an alternative variant nomenclature c.-24A>C (not HGVS), a previous report by [70] had not been taken into account for variant classification. After consultation with the author [70], correct nomenclature was confirmed, thus, the allelic data of the report was considered, strengthening the PM3 criteria, and hence, the variant pathogenicity. Final classification and comment on clinical significance for each variant was submitted to ClinVar.

**Figure 5:**
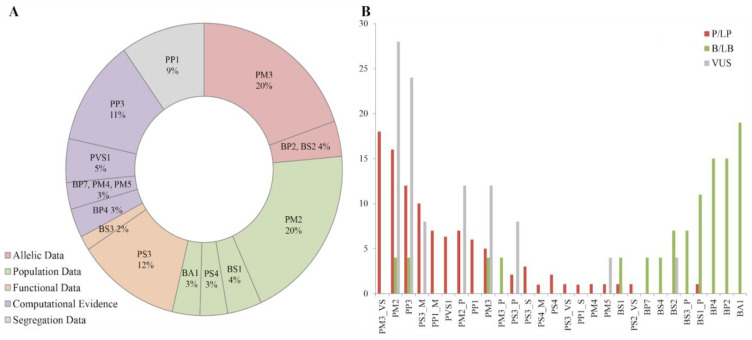
Frequency of rules applied during curation procedure. (**A**). PM2 and PM3 were the most frequently criteria applied (corresponding to population and allelic data), along with the PS3 rule, which included functional studies demonstrating a deleterious effect. (**B**). ACMG/AMP and HL-EP rules correspondence with final classification. Red, grey and green colors indicate the final variant interpretation (P/LP, B/LB and VUS) when the rule was applied. Some criteria demonstrate a bigger weight in the classification of variants. Rules applied with a modified strength are denoted by the rule followed by _P for Supporting, _M for Moderate, _S for Strong, and _VS for Very Strong.

The most frequently applied rules were PM2 and PM3 (20% each), corresponding to population and allelic data, respectively, followed by the PS3 standard, which included functional studies demonstrating a deleterious effect (12%) (Figure 5A). The frequency of rules used among the three main categories (B/LB, VUS and LP/P) is shown in Figure 5B. In this regard, PM3_VeryStrong and PM2 rules together with computational evidence suggesting a damaging impact of the mutation to the protein (PP3) and deleterious effect demonstrated by functional assays (PS3) were the most frequent criteria applied, particularly for the classification of pathogenic and likely pathogenic variants. High frequency of variants in the general populations (BA1), neutral impact predicted by in silico analysis (BP4) and allelic data (BP2) were associated with benign or likely benign categories. In the case of variants interpreted as uncertain significance, damaging computational evidence was almost always applied along with low frequency in the general population. However, this evidence was not strong enough to determine its pathogenicity. Complete information about the criteria applied and variant interpretation is detailed in Supplementary Table S3.

## 4. Discussion

Autosomal Recessive Non-Syndromic Hearing Loss (ARNSHL) is a heterogeneous condition that affects millions of individuals worldwide. Genetic variants in *GJB*2 and *GJB*6 genes are the most prevalent genetic causes of HL among several populations, and consequently, are the focus of universal newborn hearing screening programs [77,94,95]. Identifying mutations in those genes becomes crucial in molecular strategy approaches, as they provide valuable prognostic information for medical intervention. This study provides an update and extension of our previous reports and states that sequence variants in *GJB*2 and *GJB*6 genes are frequent in Argentinean patients with non-syndromic sensorineural HL [26,27,28,96].

Molecular diagnosis due to *GJB*2 and *GJB*6 variants was more successful in family cases with ARNSHL than in sporadic ones (36% vs. 15.5%). As reported previously, our data confirms c.35delG as the most frequent *GJB*2 mutation causing non-syndromic hearing loss in the Argentinean population with a prevalence of 9.25% of the detected *GJB*2-mutated alleles [26,27,28,30]. These results are in concordance with Caucasian population frequencies [73,75,87,97]. On the other hand, since 26/98 (26.5%) genotyped patients resulted in *GJB*2 compound heterozygous for non-c.35delG alleles, appropriate molecular diagnosis requires the complete sequencing of the gene including the untranslated exon 1. Interestingly, both dominant mutations in *GJB*2 were detected in the same protein residue (Arg75) and led to a profound HL phenotype, in accordance with the dominant negative effect of the two variants demonstrated by functional studies [98,99,100]. Of note, both variants del(*GJB*6-D13S1830) and del(*GJB*6-D13S1854) accounted for 15.3% of genotyped patients, which resulted in similar frequencies reported in Spain but greater than other European countries [6,7,8,21,97,101,102,103]. Our cohort of patients exhibited non-syndromic moderate-to-profound hearing loss due to biallelic *GJB*2 mutations and compound *GJB*2/*GJB*6 mutations. In accordance with previous studies truncating variants were mostly related to profound HL, while non-truncating variants to a milder degree of hearing impairment, reinforcing the notion that inactivating variants lead to a severe degree of HL [8,34,46,97].

Regarding variant curation, allelic and population data along with computational evidence were the most used information in variant assessment. The PM3 and PM2 rules accounted for 20% each of the total parameters applied. The PM3 criteria gathers the information regarding the compound heterozygous variants in HL patients. Since *GJB*2 variants are mostly related to an autosomal recessive mode of inheritance, the identification of a second mutation resulted essential and conclusive in variant interpretation. In addition, the absence or low frequency of the sequence variant in the general population (defined by PM2 rule) represented an important evidence during data analysis.

The curation of variants performed in the present work highlights the importance of specialized guidelines to analyze and interpret variants for the clinical use of databases. Moreover, it indicates the need of scientific community interaction and data sharing to avoid or reduce difficulties in variant curation [104,105]. Manual curation, although time consuming is strictly needed, as shown for the p.Met163Leu variant, which was originally interpreted as pathogenic for autosomal dominant HL by one submitter, and now reclassified to uncertain significance. An additional example is that of the splice site variant c.-22-2A>C, classified as of uncertain significance and now reinterpreted to likely pathogenic as a result of the clarification of nomenclature issues, and hence, strengthening the criteria applied. Likewise, more than half of the mutations were reclassified after the curation procedure, and satisfyingly, this reduced the number of “conflicting interpretation” categorization submitted to ClinVar.

In summary, the present work provides a set of variants that lead to hearing loss with an accurate interpretation of their phenotypic consequence. Moreover, it demonstrates the importance of a comprehensive analysis of sequence data performed by experts in the hearing field in order to provide reliable data to be used by clinicians in patient diagnosis and genetic counseling.

## Figures and Tables

**Table 1:** Curated Variants. A total of 48 sequence variants were identified in *GJB*2 and *GJB*6 and 44 were manually curated, since four variants had been already curated by the HL-EP group (with asterisk). In bold format are remarked the 27 variants evaluated in this study that changed their previous category submitted in ClinVar, based on the specific criteria applied during the curation procedure.

Nucleotide Change	Protein Variant	Mutated Alleles/ Total Alleles Tested (Percentage)	Reference	ClinVar	Final Classification (ACMG/AMP HL-EP)	Rules Applied
c.-23+1G>A	-	2/1200 (0.16%)	[68]	Pathogenic	Pathogenic	PM2, PVS1, PM3_VS, PS3_P
c.-22-12C>T	-	1/1200 (0.083%)	[69]	Benign	Benign	BA1, BP4
c.-22-2A>C	-	1/1200 (0.083%)	[70]	VUS *	**Likely Pathogenic**	PM3_M, PP1_S, PS3_P, BS1.
c.-15C>T	-	1/1200 (0.083%)	[69]	Benign/ Likely Benign	**Benign**	BA1, BP4
c.23C>T	p.Thr8Met	1/1200 (0.083%)	[71]	Conflicting Interpretation	**VUS**	PM2_P, PS3_P, PM3
c.24G>A	p.(Thr8=)	1/1200 (0.083%)	[72]	Conflicting Interpretation	**VUS**	PP3, PM2
c.29T>C	p.Leu10Pro	1/1200 (0.083%)	[73]	absent	**VUS**	PM2, PP3, PS3_M
c.35G>T	p.Gly12Val	2/1200 (0.16%)	[74]	Pathogenic/ Likely Pathogenic	**Pathogenic**	PM2_M, PM3_VS, PP3, PS3_M
c.35delG	p.Gly12Valfs*2	111/1200 (9.25%)	[75]	Pathogenic *	Pathogenic	already curated by HL-EP
c.56G>C	p.Ser19Thr	1/1200 (0.083%)	[74]	Likely Pathogenic	**Pathogenic**	PM2, PM3_VS, PP1_M, PS3_M
c.59T>C	p.Ile20Thr	4/1200 (0.34%)	[76]	Pathogenic/ Likely Pathogenic	**Pathogenic**	PM2, PM3, PP1_P, PP3, PS3_M
c.79G>A	p.Val27Ile	100/1200 (8.34%)	[77]	Benign	Benign	BA1, BP2, BS3_P
c.101T>C	p.Met34Thr	19/1200 (1.58%)	[78]	Pathogenic *	Pathogenic	already curated by HL-EP
c.109G>A	p.Val37Ile	10/1200 (0.84%)	[77]	Pathogenic *	Pathogenic	already curated by HL-EP
c.139G>T	p.(Glu47*)	6/1200 (0.5%)	[79]	Pathogenic	Pathogenic	PVS1, PM2_P, PM3_VS
c.167delT	p.Leu56Argfs*81	14/1200 (1.16%)	[75]	Pathogenic *	Pathogenic	already curated by HL-EP
c.223C>T	p.Arg75Trp	1/1200 (0.083%)	[80]	Pathogenic	Pathogenic	PM2, PS2_VS, PP1_P, PP3, PS3
c.224G>A	p.Arg75Gln	1/1200 (0.083%)	[81]	Pathogenic	Pathogenic	PM2, PS4_M, PP1_M, PM5, PP3, PS3_M
c.229T>C	p.Trp77Arg	1/1200 (0.083%)	[82]	Pathogenic	Pathogenic	PM2_P, PM3_VS, PP3, PS3_M
c.232dupG	p.(Ala78Glyfs*24)	1/1200 (0.083%)	[83]	Likely Pathogenic	**Pathogenic**	PM2, PVS1, PM3
c.232G>T	p.(Ala78Ser)	1/1200 (0.083%)	[28]	absent	**VUS**	PM2, PP3, PM5
c.246C>G	p.Ile82Met	1/1200 (0.083%)	[84]	Likely Pathogenic	**Pathogenic**	PM2, PP1_M, PM3_VS, PP3, PS3_M
c.249C>G	p.Phe83Leu	2/1200 (0.16%)	[85]	Benign/ Likely Benign	**Likely Benign**	BS1_P, BP2, BS3_P
c.250G>C	p.Val84Leu	1/1200 (0.083%)	[77]	Pathogenic/ Likely Pathogenic	**Pathogenic**	PM2, PP3, PM3_VS, PP1_P
c.269T>C	p.Leu90Pro	3/1200 (0.25%)	[68]	Conflicting Interpretation	**Pathogenic**	BS1_P, PP3, PM3_VS, PS3_M
c.269dup	p.(Val91Serfs*11)	1/1200 (0.083%)	[68]	Pathogenic	Pathogenic	PM2, PVS1, PM3_VS, PP1_M
c.283G>A	p.(Val95Met)	2/1200 (0.16%)	[77]	Pathogenic/ Likely Pathogenic	**Pathogenic**	PM2, PM3_VS, PP1_P, PP3
c.313_326del14	p.(Lys105Glyfs*5)	2/1200 (0.16%)	[79]	Pathogenic	Pathogenic	PM2_P, PVS1, PM3_VS.
c.326G>T	p.Gly109Val	1/1200 (0.083%)	[86]	absent	**Likely Pathogenic**	PM2, PM3, PS3_M
c.334_335delAA	p.(Lys112Glufs*2)	2/1200 (0.16%)	[77]	Pathogenic /Likely Pathogenic	**Pathogenic**	PM2, PVS1, PM3_VS, PP1_M
c.358_360delGAG	p.Glu120del	2/1200 (0.16%)	[79]	Pathogenic	Pathogenic	PM2_P, PM4, PM3_VS, PS3_M
c.380G>A	p.Arg127His	1/1200 (0.083%)	[87]	Conflicting Interpretation	**Benign**	BA1, BS2, BS4, PM3_P
c.384C>T	p.(Ile128=)	1/1200 (0.083%)	[75]	Likely Benign	Likely Benign	PM2, BP2, BP4, BP7
c.385G>A	p.(Glu129Lys)	1/1200 (0.083%)	[71]	Conflicting Interpretation	**VUS**	PM2, PM3
c.427C>T	p.Arg143Trp	5/1200 (0.42%)	[88]	Pathogenic	Pathogenic	PM2_P, PM3_VS, PP1_P
c.439G>A	p.(Glu147Lys)	1/1200 (0.083%)	[89]	Pathogenic/ Likely Pathogenic	**Pathogenic**	PM2, PM3_VS, PP1_Mod, PP3
c.457G>A	p.Val153Ile	3/1200 (0.25%)	[90]	Benign/ Likely Benign	**Benign**	BA1, BS2
c.478G>A	p.(Gly160Ser)	2/1200 (0.16%)	[85]	Conflicting Interpretation	**Likely Benign**	BS1_P, PP3, BP2, PM3
c.487A>G	p.Met163Val	2/1200 (0.16%)	[90]	VUS	VUS	PM2_P, PP3, PS3_M, BS2
c.487A>C	p.Met163Leu	1/1200 (0.083%)	[91]	Pathogenic	**VUS**	PM2, PS3_P
c.503A>G	p.(Lys168Arg)	5/1200 (0.42%)	[92]	Conflicting Interpretation	**VUS**	PM2_P, PP3
c.551G>C	p.Arg184Pro	4/1200 (0.34%)	[79]	Conflicting Interpretation	**Pathogenic**	PM2, PM3_VS, PP3, PS3_M
c.569T>A	p.(Val190Asp)	1/1200 (0.083%)	[28]	absent	**VUS**	PM2, PM3, PP3
c.617A>G	p.Asn206Ser	4/1200 (0.34%)	[90]	Pathogenic	Pathogenic	PM2_P, PP3, PP1_M, PM3_VS, PS3_M
c.632G>A	p.(Cys211Tyr)	1/1200 (0.083%)	[28]	absent	**Likely Pathogenic**	PM2, PM3,PP3, PP1_P
c.*1C>T (3′UTR)	-	2/1200 (0.16%)	[93]	Conflicting Interpretation	**Likely Benign**	BS1_P, BP4
del(*GJB*6-D13S1830)	-	12/1200 (1%)	[10]	Pathogenic	Pathogenic	PS3, PS4, PM2_P, PM3_VS
del(*GJB*6-D13S1854)	-	5/1200 (0.42%)	[21]	Pathogenic	Pathogenic	PM2, PS3, PS4, PM3_VS

## Supplementary Materials

The following are available online at https://www.mdpi.com/2073-4425/11/10/1233/s1, Table S1: Summary of HL-EP specifications, Table S2: Summary of genotypes detected and Table S3: Curated variants procedure.
