# Supplementary material for: GJB2 and GJB6 Genetic Variant Curation in an Argentinean Non-Syndromic Hearing-Impaired Cohort
Source: Genes (Basel). 2020 Oct 21;11(10):1233. doi: 10.3390/genes11101233 (PMC7589744; doi:10.3390/genes11101233)
Supplement: Supplementary file 1 [file genes-11-01233-s001.zip › genes-954563-supplementary/Supplementary Table S1_ Summary of HL-EP rules.docx]

Supplementary Table S1: Summary of HL-EP specifications.

|  | Benign | | Pathogenic | | | |
| --- | --- | --- | --- | --- | --- | --- |
|  | Strong | Supporting | Supporting | Moderate | Strong | Very Strong |
| Population Data | BA1 : MAF ≥ 0,5% for AR o ≥ 0,1% for AD / BS1 MAF ≥ 0,3% (AR) O ≥ 0,02% (AD) | BS1_P MAF ≥ 0,07 (AR) | PM2_P MAF: 0,07%-0,007% (AR)  PS4_P (AD) ≥ 2 probands with variants which meets PM2 | PM2 MAF ≤ 0,007% (AR), MAF ≤ 0,002% (AD)  PS4_M (AD) ≥ 6 probands with variants which meets PM2 | PS4 Fisher Exact o Chi-Squared analysis shows statistical increase in cases over controls (AR) OR (AD) ≥ 15 probands with variants which meets PM2 |  |
| Computational and predictive data |  | BP4: REVEL Score ≤ 0,15 or no impact on splicing in MaxEntScan  BP3: In-frame indels in repeat region without known function  BP7: Silent variant with no predicted impact to splicing | PP3: REVEL Score ≥ 0,7 or predicted impact to splicing using MaxEntScan | PM4: Protein length change due to an in-frame deletion or insertion that are not located in repetitive region  PM5: Novel missense change at an amino acid residue where a different pathogenic missense change was seen before | PS1: Novel nucleotide change leading to same pathogenic missense variant (splicing: intronic -12 to -1; -1 to +6)  PM5_S: Novel missense change at an amino acid residue where at least 2 different pathogenic missense changes was seen before. | PVS1: Predicted null variant by NMD in a gene where LOF is a known mechanism Disease. Guidelines for interpreting the PVS1 criteria are detailed in [Abou Tayoun et al. 2018](https://paperpile.com/c/XPLn72/u1MN) [ 57]. |
| Functional Data |  | BS3_P: Functional studies shows no deleterious effect (predefined list) | PS3_P: Functional studies with limited validation show a deleterious effect (appropriate assays) | PS3_M: Validate functional studies show a deleterious effect (predefined list)  PM1: Mutational hot spot or well-studied functional domain without benign variation (KCNQ4 pore-forming region; Gly residues in Gly-X-Y motifs of COL4A3/4/5) | PS3: Knock- in mouse model demonstrates the phenotype (exceptions in GJB2, COCH and SLC26A4) |  |
| Segregation Data | BS4: Non-segregation with disease (genotype +, phenotype -) |  | PP1: Segregation in 1 affected relative for recessive and two affected relatives for dominant | PP1_M: Segregation in 2 affected relatives for recessive and 4 affected relatives for dominant | PP1_S: Segregation in 3 affected relatives for recessive and 5 affected relatives for dominant |  |
| De Novo Data |  |  | PS2_P: 0,5 point per table 5a and 5b; Example: 1 assumed de novo occurrence (phenotype/gene not specific) | PS2_M: 1 point per table 5a and 5b; Example: 1 proven de novo occurrences (phenotype consistent but not specific to gene) or 1 assumed de novo occurrence; or 2 assumed de novo occurrences (phenotype/gene not specific) | PS2: 2 points per table 5a and 5b; Example: 1 proven de novo occurrences or 2 assumed de novo occurrences | PS2_VS: 4 points per table 5a and 5b; Example: 2 proven de novo occurrences or 1 proven + 1 assumed de novo occurrences OR 4 assumed de novo occurrences |
| Allelic Data | BS2: Observation of variant (biallelic with known pathogenic variant for recessive) in controls inconsistent with disease penetrance. | BP2: Observed in trans with a dominant variant / observed in cis with a pathogenic variants (use with caution) | PM3_P: 0,5 point awarded from tables 6a and 6b  Example: 2 variants that meet PM2_P detected in trans; OR homozygous variant meeting PM2_P | PM3: 1 point awarded from tables 6a and 6b  Example: Detected in trans with a pathogenic variant (recessive) | PM3_S: 2 points awarded from tables 6a and 6b  Example: Detected in trans in 2 probands with a pathogenic variant (recessive) | PM3_VS: 4 points awarded from tables 6a and 6b  Example: Detected in trans in ≥ 4 probands with a pathogenic variant (recessive) |
| Phenotypic Data |  | BP5: Variant in an autosomal dominant gene found in a patient with an alternate explanation | PP4: Patient's phenotype highly specific for a gene or fully sequenced gene set (see specifications table 7) |  |  |  |

Original evidence codes included a first letter P (“pathogenic”) or B (“benign”), followed by VS (“very strong”), S (“strong”), M (“moderate”), P (“supporting”), or A ( “standard alone”) to indicate the strength level and a category number. Modified codes include a suffix of an underscore and the adjusted strength level. Extensive information regarding tables 4 to 7 mentioned are detailed in the HL-EP manuscript [43].
