# Supplementary material for: GJB2 and GJB6 Genetic Variant Curation in an Argentinean Non-Syndromic Hearing-Impaired Cohort
Source: Genes (Basel). 2020 Oct 21;11(10):1233. doi: 10.3390/genes11101233 (PMC7589744; doi:10.3390/genes11101233)
Supplement: Supplementary file 1 [file genes-11-01233-s001.zip › genes-954563-supplementary/Supplementary Table S2_ Summary of genotypes detected.pdf]

#patient	Phenotype		Familial/ Sporadic
	Onset	HL severity	
1	Prelingual	Profound	Sporadic
2	Prelingual	Profound	Sporadic
3	Prelingual	Profound	Sporadic
4	Prelingual	Profound	Sporadic
5	Prelingual	Profound	Familial
6	Prelingual	Severe	Familial
7	Prelingual	Profound	Sporadic
8	Prelingual	Profound	Familial
9	Prelingual	Profound	Sporadic
10	Prelingual	Profound	Sporadic
11	Prelingual	Profound	Sporadic
12	Prelingual	Profound	Sporadic
13	Prelingual	Profound	Sporadic
14	Prelingual	Profound	Sporadic
15	Prelingual	Profound	Sporadic
16	Postlingual	Moderate	Sporadic
17	Prelingual	Profound	Sporadic
18	Prelingual	Profound	Sporadic
19	Prelingual	Profound	Sporadic
20	Prelingual	Moderate	Sporadic
21	Prelingual	Profound	Familial
22	Prelingual	Profound	Sporadic
23	Prelingual	Profound	Sporadic
24	Prelingual	Profound	Sporadic
25	Prelingual	Profound	Sporadic
26	Prelingual	Severe	Sporadic
27	Prelingual	Profound	Sporadic
28	Prelingual	Profound	Sporadic
29	Prelingual	Profound	Familial
30	Prelingual	Profound	Familial
31	Prelingual	Profound	Sporadic
32	Prelingual	Profound	Sporadic
33	Prelingual	Profound	Sporadic
34	Prelingual	Moderate	Sporadic
35	Prelingual	Moderate	Sporadic
36	Prelingual	Severe	Sporadic
37	Prelingual	Moderate	Familial
38	Prelingual	Severe	Sporadic
39	Prelingual	Moderate	Sporadic
40	Prelingual	Profound	Familial
41	Prelingual	Profound	Sporadic
42	Prelingual	Profound	Sporadic

43	Prelingual	Profound	Familial
44	Prelingual	Profound	Sporadic
45	Prelingual	Profound	Sporadic
46	Prelingual	Profound	Sporadic
47	Prelingual	Severe	Sporadic
48	Prelingual	Profound	Sporadic
49	Prelingual	Profound	Sporadic
50	Prelingual	Profound	Sporadic
51	Prelingual	Profound	Sporadic
52	Prelingual	Profound	Sporadic
53	Prelingual	Profound	Sporadic
54	Prelingual	Severe	Familial
55	Prelingual	Severe	Sporadic
56	Prelingual	Severe	Familial
57	Postlingual	Moderate	Sporadic
58	Prelingual	Profound	Sporadic
59	Prelingual	Profound	Sporadic
60	Prelingual	Severe	Familial
61	Prelingual	Profound	Familial
62	Prelingual	Moderate	Familial
63	Prelingual	Profound	Sporadic
64	Prelingual	Profound	Sporadic
65	Prelingual	Moderate	Familial
66	Prelingual	Moderate	Sporadic
67	Prelingual	Profound	Sporadic
68	Prelingual	Moderate	Familial
69	Prelingual	Profound	Familial
70	Prelingual	Profound	Familial
71	Prelingual	Moderate	Familial
72	Prelingual	Severe	Familial
73	Prelingual	Profound	Sporadic
74	Prelingual	Moderate	Sporadic
75	Prelingual	Moderate	Sporadic
76	Postlingual	Moderate	Sporadic
77	Prelingual	Profound	Sporadic
78	Prelingual	Severe	Sporadic
79	Prelingual	Profound	Sporadic
80	Prelingual	Profound	Sporadic
81	Prelingual	Profound	Sporadic
82	Prelingual	Profound	Sporadic
83	Postlingual	Moderate	Familial
84	Prelingual	Profound	Sporadic
85	Prelingual	Profound	Sporadic
86	Prelingual	Profound	Sporadic

87	Postlingual	Profound	Familial
88	Postlingual	Moderate	Sporadic
89	Prelingual	Profound	Sporadic
90	Prelingual	Profound	Sporadic
91	Postlingual	Moderate	Sporadic
92	Prelingual	Profound	Sporadic
93	Prelingual	Profound	Familial
94	Prelingual	Severe	Sporadic
95	Prelingual	Profound	Sporadic
96	Prelingual	Moderate	Sporadic
97	Postlingual	Severe	Sporadic

disease-causative gentoype detected	Family Segregation
c.35delG (;) 35delG	N/A
c.35delG (;) 35delG	N/A
p.(Glu47*) (;) del(GJB6-D13S1830)	N/A
c.35delG (;) p.Trp77Arg	N/A
[GJB2:c.35delG] ; [GJB6:del(GJB6-D13S1830)]	yes
c.35delG (;) p.Ile82Met	N/A
p.Arg75Trp (;) p.Val27Ile	N/A
c.[35delG] ; [35delG]	yes
c.[35delG] ; [-23+1G>A]	yes
c.[35delG] ; p.[Arg143Trp]	yes
c.[35delG] ; [35delG]	yes
c.[35delG] ; [35delG]	yes
c.[35delG] ; [35delG]	yes
GJB2:c.35delG (;) GJB6:del(GJB6-D13S1854)	N/A
c.35delG (;) 167delT	N/A
p.Met34Thr (;) Met34Thr	N/A
c.35delG (;) 35delG	N/A
p.[(Glu47*)] ; [Gly109Val]	yes
c.35delG (;) 35delG	N/A
p.Val37Ile (;) c.167delT	N/A
c.35delG (;) 35delG	N/A
c.35delG (;) p.(Val190Asp)	N/A
p.Arg75Gln / +	N/A
c.[35delG] ; [35delG]	yes
[GJB2:c.35delG] ; [GJB6:del(GJB6-D13S1854)]	yes
c.35delG (;) 35delG	N/A
c.35delG (;) 167delT	N/A
p.[Arg143Trp] ; c.[167delT]	yes
c.35delG (;) 35delG	N/A
35delG(;) 167delT	N/A
c.35delG (;) 35delG	N/A
GJB2:c.35delG (;) GJB6:del(GJB6-D13S1830)	N/A
c.35delG (;) 35delG	N/A
c.[313_326del14] ; p.[Glu120del]	yes
p.Val37Ile (;) (Glu47*)	N/A
p.Ile20Thr (;) Ile20Thr	N/A
c.35delG (;) p.Met34Thr	N/A
35delG (;)167delT	N/A
p.Met34Thr (;) Met34Thr	N/A
[GJB2:c.35delG] ; [GJB6:del(GJB6-D13S1830)]	yes
del(GJB6-D13S1830) (;) del(GJB6-D13S1854)	N/A
c.35delG (;) 35delG	N/A

c.[35delG] ; [35delG]	yes
[GJB2:c.35delG] ; [GJB6:del(GJB6-D13S1854)]	yes
c.35delG (;) p.(Glu47*)	N/A
del(GJB6-D13S1830);del(GJB6-D13S1830)	yes
c.35delG (;) -23+1 G>A	N/A
c.[35delG] ; [35delG]	yes
c.35delG (;) p.Arg184Pro	N/A
c.35delG (;) 35delG	N/A
c.[35delG] ; [167delT]	yes
c.35delG (;) 35delG	N/A
c.35delG (;) 35delG	N/A
p.Ile20Thr (;) Ile20Thr	N/A
c.[35delG] ; p.[Asn206Ser]	yes
c.35delG (;) 35delG	N/A
c.[35delG] ; [-22-2A>C]	yes
c.[35delG] ; [167delT]	yes
c.35delG (;) 35delG	N/A
c.[35delG] ; [269dup]	yes
c.35delG (;) 35delG	N/A
p.[(Val95Met)] ; [Gly12Val]	yes
c.[35delG] ; p.[Arg184Pro]	yes
c.[35delG] ; [35delG]	yes
c.[35delG] ; [35delG]	yes
c.35delG (;) p.Arg184Pro	N/A
[GJB2:c.35delG] ; [GJB6:del(GJB6-D13S1854)]	yes
c.[35delG] ; p.[Val37Ile]	yes
c.35delG (;) 167delT	N/A
c.[35delG] ; [35delG]	yes
c.[35delG] ; [35delG]	yes
c.[35delG] ; p.[Arg143Trp]	yes
c.35delG (;) p.Arg143Trp	N/A
c.35delG (;) 35delG	N/A
p.Val84Leu (;) c.313_326del14	N/A
p.Val37Ile (;) del(GJB6-D13S1830)	N/A
p.[Arg143Trp] ; c.[232dupG]	yes
c.[167delT] ; p.[Arg184Pro]	yes
c.35delG (;) 35delG	N/A
c.35delG (;) 35delG	N/A
[GJB2:c.35delG] ; [GJB6:del(GJB6-D13S1830)]	yes
c.35delG (;) 35delG	N/A
p.Met34Thr (;) Ser19Thr	N/A
c.35delG (;) p.(Glu47*)	N/A
GJB2:c.35delG (;) GJB6:del(GJB6-D13S1830)	N/A
p.Val37Ile (;) Gly12Val	N/A

p.[Asn206Ser] ; [Asn206Ser]	yes
p.Met34Thr (;) Met34Thr	N/A
c.35delG (;) p.Val95Met	N/A
p.(Glu47*) (;) Glu120del	N/A
p.[Cys211Tyr] ; del(GJB6-D13S1830)	yes
p.Asn206Ser (;) del(GJB6-D13S1830)	N/A
c.167delT (;) 167delT	N/A
c.35delG (;) 35delG	N/A
c.35delG(;) 167delT	N/A
p.Val37Ile (;) c.167delT	N/A
c.35delG (;) p.Leu90Pro	N/A
